# The development of the gut microbiome and temperament during infancy and early childhood: A systematic review

**DOI:** 10.1002/dev.22306

**Published:** 2022-07-13

**Authors:** Emma Alving‐Jessep, Edith Botchway, Amanda G. Wood, Anthony C. Hilton, Jacqueline M. Blissett

**Affiliations:** ^1^ Institute of Health and Neurodevelopment, College of Health and Life Sciences Aston University Birmingham UK; ^2^ School of Psychology Deakin University Burwood Victoria Australia

**Keywords:** child, development, gut microbiome, gut–brain axis, infant, temperament

## Abstract

Temperament in early childhood is a good predictor of later personality, behavior, and risk of psychopathology. Variation in temperament can be explained by environmental and biological factors. One biological mechanism of interest is the gut microbiome (GM), which has been associated with mental and physical health. This review synthesized existing literature evaluating the relationship between GM composition and diversity, and temperament in early life. Web of Science, PsycInfo, PubMed, and Scopus were searched, and data were extracted according to PRISMA guidelines. In total, 1562 studies were identified, of which six remained following application of exclusion/inclusion criteria. The findings suggest that there is an association between higher alpha diversity and temperament: greater Surgency/Extraversion and High‐Intensity Pleasure in males, and lower Effortful Control in females. Unique community structures (beta diversity) were found for Surgency/Extraversion in males and Fear in females. An emerging pattern of positive temperament traits being associated with GM communities biased toward short‐chain fatty acid production from a metabolism based on dietary fiber and complex carbohydrates was observed and is worthy of further investigation. To gain deeper understanding of the relationship, future research should investigate further the functional aspects of the microbiome and the influence of diet.

## 1 INTRODUCTION

The global prevalence of mental health disorders in children and adolescents, aged between 6 and 18 years, is close to 15% (Polanczyk et al., 2015). For example, it is estimated that currently in the United Kingdom, one in eight children aged between 5 and 19 years old is diagnosed with at least one mental health problem, including emotional, behavioral, and/or hyperactivity disorders (Baker, 2021). These disorders typically have a pervasive effect upon individuals throughout their childhood, adolescence, and into adulthood (Kretschmer et al., 2014). Identification of early childhood markers of later behavioral and mental health problems provides the possibility of developing interventions and prevention programs targeted at early life stages. Measures of temperament in early childhood, through parent, teacher, or observational measures, have been shown to be good predictors of personality, behavior, and risk of psychopathology in later childhood and adolescence (Muris & Ollendick, 2005), and thus are good early targets for investigation of preceding markers.

Temperament, which refers to an individual's patterns of behavior, including emotional responsiveness, mood, and the speed and intensity of reactions, is often considered to be a fundamental component of personality, present early in life (Sanson et al., 2004). During early childhood, an individual's reactions to their environment are predominantly influenced by temperament (Rothbart, 2012) and temperamental traits in children are closely linked with the broad factors used to describe personality traits in adulthood (McCrae et al., 2000). Temperament in childhood can give insight into later behavior through its close relationship with personality (Rothbart et al., 2011), suggesting that it is a strong marker of later behavioral phenotypes. Although temperamental traits have previously been considered to be stable over time, it is possible for them to undergo change during an individual's development (Rothbart & Bates, 2006). Evidence for individual differences in temperament has shown that between 20% and 60% of phenotypical variance in personality can be accounted for by genetics (Saudino, 2005). Nevertheless, data from twin and adoption studies have also shown that environmental factors play an important role in individual differences in child temperament (Saudino, 2005).

Composition of the gut microbiome (GM) is likely to influence children's temperament, given that several studies in children have implicated the GM in a range of other physical and mental and developmental outcomes. The GM includes both the composition of the communities of bacteria, viruses, archaea, and fungi that colonize the gut, as well as the collective genome. In contrast, the term “microbiota” refers to the composition of a community including bacteria, viruses, archaea, and fungi but not its collective genome. In this review, the use of GM refers exclusively to gut microbiome, and where microbiota is the topic of discussion, this term is written in full.

Studies in children have implicated the GM in several health outcomes, including physical health conditions, such as obesity (Murugesan et al., 2018) and asthma (Attar, 2015; Moossavi et al., 2018), as well as mental health conditions such as attention deficit hyperactivity disorder (ADHD; Adesman et al., 2017). Furthermore, bacterial colonization of the gut has been shown to be directly related to the maturation of both the central nervous system (CNS) and enteric nervous system (ENS) in children (Barbara et al., 2005; Stilling et al., 2014). From a developmental perspective, the most rapid phase of colonization of the gut starts at birth and continues until maturation of the GM at approximately 31–46 months (Stewart et al., 2018). The GM and brain are thought to share similar sensitive periods of development during infancy that are known to extend up until the second year of life (Borre et al., 2014; Heijtz et al., 2011). Sensitive periods in the development of the microbiota include birth and the early postnatal period, as well as during complementary feeding (the period of introduction to solid food, typically around 6 months of age as recommended by the World Health Organization [WHO]). These periods align with neurodevelopmental periods of plasticity including sensory function, language, learning, and memory (for a review, see Cowan et al., 2020).

The GM and brain share a bidirectional relationship, and this communication route between them is known as the gut–brain axis (GBA; Wang et al., 2018). The GBA comprises several pathways including the CNS, ENS, and the hypothalamic–pituitary–adrenal axis (HPA; Skonieczna‐Żydecka et al., 2018). Through their metabolism of several substrates including dietary fiber and carbohydrates, bacteria produce short‐chain fatty acids (SCFA) consisting primarily of acetate, propionate, and butyrate (Silva et al., 2020). Each bacterium can be categorized by the SCFA that they produce, and each may produce one or more, through different metabolic processes (Louis & Flint, 2017). Production of SCFAs in the gut plays an important role in maintaining gut health, including prevention of inflammation and maintenance of intestinal barrier function. SCFAs additionally play a central role in the communication in the GBA (Silva et al., 2020). Bacteria within the gut also play an important role in the metabolism of tryptophan, an amino acid precursor of serotonin production, with the serotonergic system being key to the regulation of mood (Jenkins et al., 2016). For these reasons, altered composition of the GM, through colonization of aberrant species or changes in overall diversity or composition, may disrupt the communication of the GBA and further impact both physical and mental health of an individual. It is plausible that these effects may be evident in early development of temperament.

Animal models provide further evidence for the mechanism by which GM may influence the development of temperament. Studies of germ‐free (GF) animals (specially raised animals that are free from all microorganisms) have shown that dysregulation of the GM is associated with lasting impact upon brain chemistry affecting stress response, cognition, and behavior relating to anxiety and depression (Cryan & Dinan, 2012; Zheng et al., 2016). In humans, the HPA axis (which plays an important role in emotional regulation and stress response) has been shown to be affected by GM dysregulation (De Weerth, 2017). It is plausible that these changes in the composition of the GM may alter both the functioning of the HPA axis and the relationship between the ENS and the CNS, which has been suggested as a mechanism that drives individual differences in temperament (Luczynski et al., 2016).

To understand how variation in temperament is related to later adverse development, it is important to understand the relationship between temperament and its underpinning biological mechanisms, specifically the development of the GM. Despite emerging and developing interest in the relationship between the GM and temperament, there is no current consensus regarding specific bacterial composition or diversity of the GM and its relationship with different aspects of temperament. Thus, the aim of this systematic review was to gather and synthesize existing evidence relating to the relationship between GM composition and diversity and temperament in early childhood.

## 2 METHODS

A systematic review was conducted using methods set out in the Preferred Reporting Items for Systematic Review and Meta‐Analysis (PRISMA) guidelines (Moher et al., 2010). A protocol for this systematic review was registered on PROSPERO, registration number CRD42020196919.

### 2.1 Data sources

A search of the academic databases Web of Science, PsycInfo, PubMed, and Scopus was conducted from September to October 2020. Search terms were established relating to gut (gut, intestin*, enterotype), microbiome (microbiome, microbio* or bacteri*, composition, diversity), and temperament (temperament, personality, anxiety, sociability, “negative affect,” fear, shyness, mood, stress). Following establishment of search terms, Boolean operators were applied, and appropriate adjustments were made for each database. This process was repeated in June 2021 to ensure the most up‐to‐date papers were included.

### 2.2 Eligibility criteria

All studies reporting on the relationship between variation in composition of GM and temperament in children up to the age of 6 years 11 months were eligible for inclusion. From the age of 7 years, the temperament measures used move toward the middle and late childhood, 8–14 years of age. Therefore, for the purpose of this review the age range was limited to the early childhood years and related measurements. The study types of interest were cross‐sectional and longitudinal observational studies. Inclusion criteria for this review were as follows:

Studies that measured the composition and/or diversity of GM using either whole genome sequencing, 16S rRNA, or shotgun sequencing methods;

Studies that used established scales to measure behavioral outcome measures of temperament;

Studies involving healthy child participants (and their parents), from birth to 6 years 11 months;

Studies involving children born at full term, after 36 weeks but before 42 weeks;

Studies with children born either via vaginal delivery or caesarean section;

Studies involving children with either single or multiple births, with or without siblings.

*Exclusion criteria for this review were as follows*:

Studies not written in English;

Reviews, meta‐analyses, book sections, book chapters, and studies not published in peer‐reviewed journals;

Studies that did not measure the outcomes of interest and/or did not use next‐generation sequencing to measure GM composition;

Studies with children born prematurely <36‐week gestation or late >42‐week gestation;

Studies of children with diagnosed gastrointestinal health conditions, such as studies focused on Crohn's disease;

Studies focused on children with severe/multiple allergies;

Studies of children with diagnosed genetic disorder or syndrome, learning or developmental disorder, or acquired injuries that have known links with altered GM composition or behavior, such as studies focused on autism.

### 2.3 Study selection and data extraction

Endnote version x9 software was used to collate articles from academic database searches and to remove duplicate articles. Two reviewers, EAJ and EB, independently screened each article by title and abstract to remove articles that either did not meet the inclusion criteria or met the exclusion criteria. Full articles were then independently screened for inclusion by two reviewers (EAJ and EB), and final selection was made for data extraction. A third reviewer (JB) was consulted to settle discrepancies in review decisions at each stage of the review process. Researcher EAJ extracted all data for the final articles included in this review. Data were first extracted into an excel sheet for synthesis, including details on author and location of study, publication year, study design, participant demographics including age at each time point, GM technique including, collection method, sequencing method, hypervariable regions, temperament measurement, GM diversity, and/or composition measures. Statistical methods and study results were also extracted including significance levels and effect sizes with confidence intervals, where possible.

### 2.4 Quality assessment

All articles that met inclusion criteria for this review were assessed for risk of bias independently by two reviewers (EAJ and EB). The National Heart, Lung, and Blood Institute (NHLBI) quality assessment tool for observational cohort and cross‐sectional studies (NHLBI, 2021) was used to rate the articles. This tool has 14 questions evaluating the inclusion and quality of the research question, study population, sample size justification, exposure measures, outcome measures, statistical analyses, timeframe, blinding, and repeated exposures. Each question was scored as either “yes,” where the criteria is satisfied; “no,” where the criteria is not met; or “not applicable.” A score of “yes” corresponded with one and “no” or “not applicable” corresponded with zero. Scores for each article were totaled, and a grading system developed by Uloko et al. (2018) was employed to rate the selected articles into: “Good” (≥70%), “Fair” (≥50%), and “Poor” (<50%).

## 3 RESULTS

**FIGURE 1:**
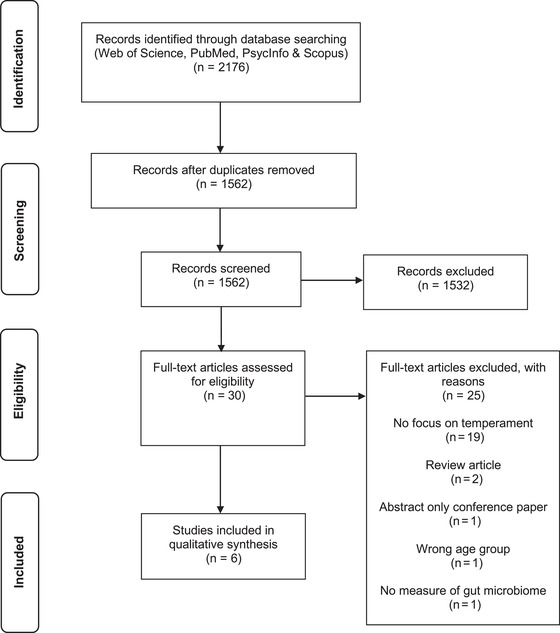
PRISMA flowchart illustrating systematic review screening process

A total of 2176 articles were identified (1698 in the first search [S1], 478 in the second search [S2]). Duplicate articles (*n* = 614: S1 = 544, S2 = 70) were removed, and 1562 (S1 = 1154, S2 = 408) articles were screened by abstract and title. Following the first screening, 1128 did not meet the eligibility criteria and were removed. Full text screening was carried out on a total of 30 articles (S1 = 26, S2 = 4) and 24 articles were unanimously excluded, including one for which the third reviewer (JB) was consulted to resolve conflict in the review decision. Six articles were included in this systematic review, each reporting on a unique study, that met the review criteria as shown in Figure 1, PRISMA flowchart (Moher et al., 2010).

### 3.1 Study characteristics

The six studies included a total of 733 participants, with individual studies including 40–301 participants (Aatsinki et al., 2019; Christian et al., 2015; Flannery et al., 2020; Kelsey et al., 2021; Loughman et al., 2020; Wang et al., 2020). Child participants ranged in age from birth to 6 years 11 months old. Publication dates of the articles ranged from 2015 to 2021 (see Table 1). Two studies were longitudinal studies (Aatsinki et al., 2019; Loughman et al., 2020) and four were cross‐sectional observational studies. Both longitudinal studies recruited families during the prenatal period and have continuing follow‐ups, one focused on the age range from birth to 6 months of age, and the other from birth to 2 years of age. Location of study samples occurred across several continents; three were conducted in the United States, one in Australia, one in Europe (Finland), and one in China.

**TABLE 1:** Summary of article characteristics (*N* = 6)

Authors	Country	Number of participants	Age range child	Average age parents (if given)	Ethnicity	SES proxy markers
1. Christian et al. (2015)	United States	77 mother–toddler dyads	18–27 months of age, mean = 23.14 (*SD* = 2.00)	Maternal mean = 31.1 (*SD* = 5.43)	87% (*n* = 67) White, 9.1% (*n* = 7) Black, 3.9% (*n* = 3) Asian	None given.
2. Aatsinki et al. (2019)	Finland	Subcohort of 301 (159 boys, 142 girls)^a^	From birth followed until 6 months of age	Mother mean = 30.8 (*SD* = 4.3)	Not specified	Maternal level of education reported. Upper secondary: *n* = 67 (22.3%) Vocational school: *n* = 97 (32.2%) Tertiary education: *n* = 128 (42.5%) NA: *n* = 9 (3%)
3. Loughman et al. (2020)	Australia	Subcohort of 201^b^	From birth followed until 2 years of age	Maternal mean = 32.4 (*SD* = 4.2) Paternal mean = 34.2 (*SD* = 5.1) In subcohort	Not specified	Socio‐Economic Indexes for Areas (SEIFA) – Australia: Low – *n* = 59 (29.4%) Middle – *n* = 73 (36.3%) High – *n* = 69 (34.3%) Unknown – *n* = 0

Authors	Country	Number of participants	Age range child	Average age parents (if given)	Ethnicity	SES proxy markers
4. Wang et al. (2020)	China	51 mother infant dyads	Mean age 12.3 months (*SD* = 0.25).	Maternal mean = 32.3 (*SD* = 4.51)	Chinese	Maternal level of education reported.
5. Flannery et al. (2020)	United States	40 mothers and infants recruited	5–7 years of age Mean age = 6.12 (*SD* = 0.69)	Not given	55.26% (*n* = 21) Caucasian; 18.42% (*n* = 7) mixed race; 18.42% (*n* = 7) Hispanic/Latinx; 7.89% (*n* = 2) Native American/American Indian	Socioeconomic risk was indexed using the socioeconomic status and the Life Events Checklist.
6. Kelsey et al. (2021)	United States	63 participants took part 23 participants were excluded due to data quality	Mean average: 24 days Range: 9–56 days	Not given	Measured as White (73%) and non‐White.	Measured as household income: <$15,000 = 5 (8%) $15,001–$30,000 = 5 (8%) $30,001–$45,000 = 3 (5%) $45,001–$60,000 = 1 (2%) $60,001–$75,000 = 2 (3%) $75,001–$90,000 = 9 (15%) $90,001–$110,000 = 7 (11%) $110,001–$125,000 = 7 (11%) $125,001–$175,000 = 2 (3%) $175,001–$225,000 = 8 (13%) $225,001–$275,000 = 8 (13%) >$275,001 = 3 (5%)

^a^ Main cohort is part of the FinnBrain Birth Cohort Study (Karlsson et al., 2018) with a total of 5790 participating families.

^b^ Main cohort is part of the Barwon Infant Study (Vuillermin et al., 2015), with a total of 1074 participants.

### 3.2 Quality assessment

Overall, the quality of articles was varied: two papers were assessed as “Good,” three “Fair,” and one “Poor” (Flannery et al., 2020; see Table 2). All articles included a clear research question, well‐defined exposures, including levels of measures, and outcome measures. Only one of the articles included in this review presented effect sizes, which satisfied question 5: “Was a sample size justification, power descriptions, or variance and effect estimates provided?” (Kelsey et al., 2021). None of the articles included power descriptions or sample size justifications. For all studies, blinding of the assessor to the exposures of participants was marked as not applicable; no studies were interventions. Cross‐sectional studies were scored as “no” to the following questions: question 6, “For the analyses in this paper, were the exposure(s) of interest measured prior to the outcome(s) being measured?”; question 7, “Was the timeframe sufficient so that one could reasonably expect to see an association between exposure and outcome if it existed?”; question 10, “Was the exposure(s) assessed more than once over time?”; and question 13, “Was loss to follow‐up after baseline 20% or less?” This resulted in lower overall quality scores for all cross‐sectional designs. The scoring was performed in line with the instructions of the NHLBI quality assessment tool for observational cohort and cross‐sectional studies (NHLBI, 2021) while acknowledging the systematic impact of the scoring system on ratings of cross‐sectional designs.

**TABLE 2:** Quality assessment scores according to the NHLBI quality assessment tool for observational cohort and cross‐sectional studies^a^

Study	Q1	Q2	Q3	Q4	Q5	Q6	Q7	Q8	Q9	Q10	Q11	Q12	Q13	Q14	Quality rating
1. Christian et al. (2015)	Yes	Yes	Yes	Yes	No	No	No	Yes	Yes	No	Yes	NA	No	No	7/14 (Fair)
2. Aatsinki et al. (2019)	Yes	Yes	Yes	Yes	No	Yes	Yes	Yes	Yes	No	Yes	NA	Yes	Yes	11/14 (Good)
3. Loughman et al. (2020)	Yes	Yes	Yes	Yes	No	Yes	Yes	Yes	Yes	Yes	Yes	NA	Yes	Yes	12/14 (Good)
4. Wang et al. (2020)	Yes	Yes	Yes	Yes	No	No	No	Yes	Yes	No	Yes	NA	Yes	Yes	9/14 (Fair)
5. Flannery et al. (2020)	Yes	No	CD	CD	No	No	No	Yes	Yes	No	Yes	NA	No	Yes	5/14 (Poor)
6. Kelsey et al. (2021)	Yes	Yes	Yes	Yes	No	No	No	Yes	Yes	No	Yes	NA	No	Yes	8/14 (Fair)

*Note*: Each question is answered either Yes, No, Cannot Determine (CD), Not Applicable (NA), or Not Reported (NR) as per guidance provided with this quality assessment tool.

^a^ Criteria questions: 1. Was the research question or objective in this paper clearly stater? 2. Was the study population clearly specified and defined? 3. Was the participation rate of eligible persons at least 50%. 4. Were all the subjects selected or recruited from the same or similar populations (including the same period)? Were inclusion and exclusion criteria for being in the study prespecified and applied uniformly to all participants? 5. Was a sample size justification, power description, or variance and effect estimates provided? 6. For the analyses in this paper, were the exposure(s) of interest measured prior to the outcome(s) being measured? 7. Was the timeframe sufficient so that one could reasonably expect to see an association between exposure and outcome if it existed? 8. For exposures that can vary in amount or level, did the study examine different levels of the exposure as related to outcome (e.g., categories of exposure, or exposure measured as continuous variable)? 9. Were the exposure measures (independent variables) clearly defined, valid, reliable, and implemented consistently across all study participants? 10. Was the exposure(s) assessed more than once over time? 11. Were the outcome measures (dependent variables) clearly defined, valid, reliable, and implemented consistently across all study participants? 12. Were the outcome assessors blinded to the exposure status of the participants? 13. Was loss to follow‐up after baseline 20% or less? 14. Were key potential confounding variables measured and adjusted statistically for their impact on the relationship between exposures(s) and outcome(s)?

### 3.3 Microbiome analyses

All the studies included in this review investigated the composition of the microbiota; Studies 5 and 6 additionally investigated the functional composition of the GM. GM diversity was assessed in all studies, except Study 4. Alpha diversity was measured in Studies 1, 2, 3, and 6, Beta diversity was measured in Studies 1 and 3, and functional beta diversity was measured in Studies 5 and 6. Four of the studies included in this review used 16S rRNA sequencing to investigate the gut microbiota, and two studies (Studies 5 and 6) used shotgun metagenomics to investigate the microbiome. Three of the 16S rRNA studies used the Illumina MiSeq platform to sequence the data, except Study 1, which used Roche 454 FLX Titanium system. Studies investigating the 16S rRNA varied in hypervariable region selection. Studies 2 and 3 used V4 region only, Study 4 used both V3 and V4, and Study 1 used V1–3 (see Table 3). Both the SILVA taxonomic data base and the GreenGenes reference database were used to identify genera of bacteria from the Operational Taxonomic Units (OTUs) assigned. Flannery et al. (2020) prepared the raw sequences for analysis using the Shot Cleaner Workflow, following the guidelines laid out by the Human Microbiome Project Consortium (2012). Furthermore, Flannery et al. (2020) used Shotmap to quantify group relative abundance by function using the Kyoto Encyclopedia of Genes and Genomes (KEGG) group relative abundance by function (Sharpton, 2017) and Metaphlan2 (Truong et al., 2015) was used to quantify taxon relative abundance. Kelsey et al. (2021) used a series of pipelines developed in‐house to analyze their microbiome samples. The JAMSalpha pipeline was used to obtain taxonomic and functional relative abundances. Metagenomic contigs were taxonomically classified using the k‐mer analysis in Kraken 2.

**TABLE 3:** Summary of key measures included in all articles (*N* = 6)

Authors	Microbiome sequencing technique	Hyper variable region	Fresh/chilled/frozen samples	Age(s) of microbiome measurement	Temperament measure	Time points and age of temperament measurement
1. Christian et al. (2015)	16S rRNA sequencing conducted on the Roche 454 FLX Titanium System PyNAST was used for sequence alignment with the GreenGenes core reference database	Primers 27F/519R were used to extract V1–3 hypervariable regions	Samples refrigerated at home +4°C. Samples were transported on ice to lab (temperature not specified) and stored at −80°C until pyrosequencing conducted.	Collected once at 18–27 months of age	Early Childhood Behavior Questionnaire. Negative affectivity, Surgency/Extraversion, and Effortful Control are measured.	Collected once at 18–27 months of age
2. Aatsinki et al. (2019)	16S rRNA sequencing (Illumina MiSeq, Qiime v1.9) was used to check sequences against GreenGenes database and annotate the OTUs	V4 region selected.	Samples were chilled by participants at +4°C	Collected once at 2.5 months of age	Infant Behavior Questionnaire—Revised Short Form (IBQ‐R SF) Cronbach's alpha across subscales ranged from 0.65 to 0.84	Collected once at 6 months of age
3. Loughman et al. (2020)	16S rRNA sequencing (Illumina MiSeq Platform, Mothur software) was used to assign taxa using SILVA v123 Nr99 taxonomic database. Samples with fewer than 2500 reads were excluded	V4 region selected.	Stool samples stored at −80°C, analysis adjustment of samples stored in home freezer (typically −18°C) prior to delivery to the laboratory.	Three times total at 1, 6, and 12 months of age.	Child behavior checklist (Achenbach, 1999) and temperament measured at 1, 6, and 12 months, using a 5‐point Likert scale	Collected once at 2 years of age
4. Wang et al. (2020)	16S rRNA sequencing Illumina MiSeq. Taxa of each sequence was analyzed using RDP classifier algorithm, against SILVA database using confidence interval at 70%.	V3 and V4 hypervariable regions were selected	Chilled in a cooler during transport +4°C, frozen until analysis at −80°C.	Collected once at 12 months of age	Infant Behavior Questionnaire—Revised (IBQ‐R) Chinese Version.	Collected once at 12 months of age
5. Flannery et al. (2020)	Shotgun metagenomic sequencing was conducted. Raw metagenomic sequences were prepared for analysis using the Shotcleaner workflow, following the Human Microbiome Project Consortium data processing guidelines. Group relative abundance, by function, was assigned using the Kyoto Encyclopedia of Genes and Genomes. Metaphlan2 was used to quantify taxon relative abundance.	NA – Shotgun metagenome measured.	Collected at ambient temperatures and then stored at −80°C until analysis.	Collected once at 5–7 years of age	The Child Behavior Questionnaire and the Child Behavior Checklist (Achenbach, 1999).	Collected once at 5–7 years of age
Table 3 continued						
Authors	Microbiome sequencing technique	Hyper variable region	Fresh/chilled/frozen samples	Time points and age of microbiome measurement	Temperament measure	Time points and age of temperament measurement
6. Kelsey et al. (2021)	Shotgun metagenomic sequencing was conducted. A series of pipelines was used developed in‐house using the R language. The JAMSalpha pipeline was used to obtain taxonomic and functional relative abundances. Kraken2 and k‐mer analyses were used to classify taxonomy.	NA – Shotgun metagenome measured.	Samples reached the lab within 24 h of the study visit—average 7.96 h. Samples were aliquoted into cryovials containing 20% glycerol and 80% phosphate‐buffered saline solution. Samples were then frozen at −80°C.	Once ranging from 9 to 56 days of life.	Infant Behavior Questionnaire—Revised Short Form (IBQ‐R SF)	Once at the same time as the microbiome sample ranging from 9 to 56 days of life.

Sample collection methods also varied, with two of the studies (1 and 2) requesting that participants stored the samples chilled at +4°C until collection/drop off at the laboratory. Studies 1, 2, and 6 requested that samples were brought to the laboratory within a 24‐h window. Studies 5 and 6 collected samples at ambient room temperature, and Study 3 collected samples either fresh or chilled in a home freezer at typically −18°C. Study 3 collected samples at time of the home visit for completion of the temperament scale; samples were transported in a cooler at +4°C for an average of 1.5 h until they reached the lab. Following collection, all but Study 2 froze their samples at −80°C until DNA extraction was performed ready for analysis. Study 2 began DNA extraction as soon as samples reached the lab.

### 3.4 Temperament measures

Temperament was typically measured using several well‐established scales (see Table 3). Study 1 used the Early Childhood Behavior Questionnaire (ECBQ; Putnam et al., 2006), Studies 2 and 6 used the Infant Behavior Questionnaire—Revised Short Form (IBQ‐R SF), Study 4 used the Chinese version of the Infant Behavior Questionnaire—Revised (IBQ‐R; Putnam et al., 2014), and Study 5 used the Child Behavior Questionnaire (CBQ; Rothbart et al., 2001). In addition, Study 3 used a single 5‐point Likert scale to measure “temperament” developed by Ponsonby et al. (1997). This scale has not been validated as an in‐depth measure of temperament in infants.

### 3.5 Microbiome and temperament outcomes

#### 3.5.1 Microbiome diversity

Alpha diversity, a measure of the diversity of species within a given ecosystem or environment, was measured in four studies (Studies 1, 2, 3, and 6). Study 1 used a phylogenetic diversity measurement, PD_whole_tree4, and the Shannon Diversity Index (SDI). In this study, microbiota measures were investigated separately for boys and girls. There were significant associations between alpha diversity, measured using phylogenetic diversity, and higher scores on the Surgency/Extraversion subscale, in both boys and girls aged between 18 and 27 months. There was no significant relationship between SDI and Surgency/Extraversion for either boys or girls. In boys, High‐Intensity Pleasure was positively associated with both phylogenetic diversity and SDI, whereas in girls Effortful Control was negatively associated with SDI but not phylogenetic diversity. Study 2 found that alpha diversity scores of SDI and Chao1, measured at 2.5 months of life, had no statistically significant associations with temperament measured at 6 months of life. Study 2 also included several covariates in addition to gender, including gestational age, infant age, mode of delivery, breastfeeding status, and antibiotic intake age. In the adjusted models, alpha diversity was associated with negative emotionality and fear reactivity. The Chao1 measure of richness was not associated with temperament in the adjusted models. Although Study 3 included alpha diversity measures, taken at 1, 6, and 12 months, the relationship between alpha diversity and temperament was not assessed. Study 6 found no significant associations between the behavioral temperament measures of negative emotionality, regulation/orienting, and surgency/positive emotionality, and alpha diversity in children aged 9–56 days (*M* = 24 days). This was consistent across all measures of alpha diversity; both taxa diversity measures, Shannon‐taxa and Chao1‐taxa; and Chao1 functional terms—diversity, virulence factors, resistome, and Gene Ontology (GO) terms. Virulence factors include the cellular structures that allow bacteria to invade and colonize a host, suppress immunity, and divert nutrition from the host. Resistome diversity is the genes within bacteria that code for products that increase resistance to antibiotics. GO terms characterize the contribution of individual genes to the biological makeup of an organism. They did, however, find significant indirect effects, which suggested that the relationship between taxa diversity and negative emotionality may be mediated by homologous–interhemispheric connectivity. Similarly, they also found significant indirect effects for virulence factors diversity and both negative emotionality and regulation/orienting, when mediated by homologous–interhemispheric connectivity. Studies 4 and 5 did not measure alpha diversity.

**FIGURE 2:**
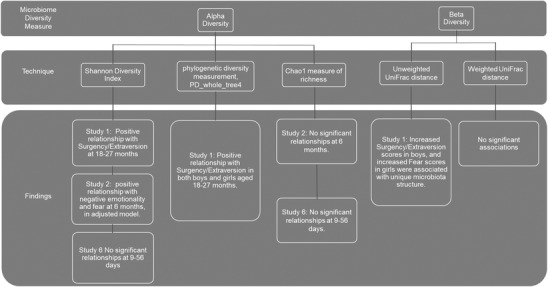
Synthesis figure illustrating the relationship between alpha and beta diversity and temperament for each study

Beta diversity, measured using weighted and unweighted UniFrac distances, is a measure of amount of compositional difference between communities or environments. Study 1 investigated beta diversity in 18–27‐month‐old children. Using the Adonis statistic, they found that Surgency/Extraversion was associated with a unique microbiota structure measured on unweighted UniFrac, but not weighted distances, in boys. Subscale analysis highlighted three subscales, Sociability, High‐Intensity Pleasure, and Activity Levels, which drive the effect seen with unweighted differences. In girls, only one subscale, Fear, was associated with a unique community structure, measured using unweighted UniFrac distances. Study 1 was the only study to investigate beta diversity related to temperament. Additionally, due to beta diversity being an index of the unique community structures of microbiota within a study population, these results are difficult to generalize further beyond that specific study population, and therefore further work is needed to establish the importance of beta diversity in the development of temperament. Figure 2 illustrates the relationship between alpha and beta diversity and temperament of all included studies.

#### 3.5.2 Microbiome composition

**FIGURE 3:**
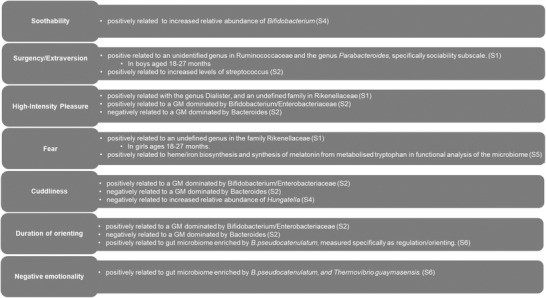
Synthesis figure illustrating the relationship between temperament and microbiota composition for each study. The numbers in brackets refer to the study number to which the results belong.

Several approaches were employed to look at the association of microbiota composition with temperament (see Table 4). Study 1 examined genera that made up at least 1% of the total sample by relative abundance, in children aged between 18 and 27 months, which was done in order to focus on the dominant, highly abundant genera (Christian et al., 2015). This included the top 20 and 18 genera in boys and girls, respectively. Notably, temperament subscales loading onto the Surgency/Extraversion composite scale were found to correlate with specific genus abundance for boys. Sociability was positively associated with an undefined genus in the Ruminococcaceae family and the genus *Parabacteroides* (see Figure 3 for synthesis of composition results). The genus *Dialister* and an unidentified genus in the family Rikenellaceae were positively associated with both High‐Intensity Pleasure and activity level. Interestingly, in girls, fear was positively associated with an unidentified genus in the family Rikenellaceae. Study 4 established that the abundance of *Bifidobacterium* was positively related to soothability, and *Hungatella* was negatively correlated to cuddliness. This relationship was controlled for delivery mode, feeding type (breast or formula), and probiotic consumption.

**TABLE 4:** Summary of key findings and limitations across all included articles (*N* = 6)

Authors	Microbiome diversity measures	Microbiome composition measures	Main results	Limitations
1. Christian et al. (2015)	Alpha diversity measured with phylogenetic diversity measurement, PD_Whole_tree, and the Shannon Diversity Index (SDI). Beta Diversity was measured using both unweighted and weighted UniFrac distances.	Genus abundances were used.	1. Showed significant sex differences in temperament ratings, including higher scores for boys in motor ratings and High‐Intensity Pleasure, while girls had higher rating of inhibitory control and soothability. 2. Among boys, higher scores of Surgency/Extraversion were associated with greater phylogenetic diversity, but not associated with Shannon Diversity Index (SDI). High‐Intensity Pleasure was associated with both greater phylogenetic diversity and SDI. 3. Surgency/Extraversion was associated with greater phylogenetic diversity, in girls, but not associated with SDI. There were also significant negative associations between the composite scores of Effortful Control and SDI, but not phylogenetic diversity. 4. In boys, sociability was positively associated with the abundances of an undefined genus in the family Ruminococcaceae and Parabacteroides. High‐Intensity Pleasure was positively associated with the genus *Dialister*, and an undefined family in Rikenellaceae. In girls, fear was positively associated with an undefined genus in the family Rikenellaceae.	1. This study was cross‐sectional and observational in approach and does not allow for the determination of causal direction of effects. 2. In this study, it was not possible to look at microbial function, as it would require a metagenomic or metatranscriptomic approach.
2. Aatsinki et al. (2019)	Alpha – Shannon Index, Chao1.	OTU counts were used for composition measures.	1. Three distinct clusters were identified. 2. Clusters and infant temperament – Bifidobacterium/Enterobacteriaceae presented the highest scores and Bacteroides cluster the lowest scores in trait of regulation (Kruskal–Wallis *H* test *χ* ^2^ = 5.8, FDR = 0.23), and the subscales of High‐Intensity Pleasure, cuddliness, and duration of orienting. 3. Alpha diversity and infant temperament – Neither Shannon Index nor Chao1 were associated with any of the temperament traits. When adjusted for gestational age, infant age, sex, mode of delivery, breastfeeding, and antibiotic intake, diversity was associated with negative emotionality (*B* = −0.17, FDR, = 0.17, adjusted *R* ^2^ = 0.016), and fear reactivity (*B* = 0.27, FDR = 0.17, adjusted *R* ^2^ = .032) Chao, richness, was not associated after adjustment. 4. Genus‐level investigation revealed that the temperament trait surgency is positively associated with streptococcus and regulation is positively associated with Erwinia, after controlling for sex, mode of delivery, infant age and gestational age, antibiotic treatment, and breastfeeding status.	1. Temperament assessment was based on report by mothers, which may be influenced by her own temperament and other characteristics. 2. Both GMC and temperament were assessed only at single time points. Serial and concurrent measurements should be undertaken in the future. 3. 16S rRNA offers comprehensive taxonomic profiling, but other methods such as shotgun sequencing could offer better resolution.
3. Loughman et al. (2020)	Alpha – Shannon, Simpson, Chao1, and observed species indices. Beta diversity both weighted and unweighted UniFrac distance.	Voom method from the limma package was used for differential normalized abundance testing.	1. No evidence of associations between 1‐ or 6‐month alpha or beta diversity and behavioral outcome measured at 2 years. 2. No differential normalized abundance in microbiota of 1‐month old associated with either behavioral case versus non‐case. 3. In 6‐month fecal samples, *Sutterella* appeared lower in the case group but was attenuated for following adjustment for storage. 4. 12‐month‐olds Alpha diversity showed weak evidence of increased risk of elevated behavioral problems (OR: 2.42 [0.92–6.97], *p* = .087). 5. PERMANOVA analysis of unweighted UniFrac distances suggested differences in microbiota community structure between groups (*R* ^2^ = .0092, *p* = .018). 6. Normalized abundance of two bacterial groups was substantially different in the 12‐month‐old behavioral case infants. *Prevotella* was detected in only 4% of the cases versus 44% of non‐case infants (logFC = −1.46, *p* < .0001, *q* < .0001). Lachnospiraceae was detected in 91% of case infants versus 69% of non‐case infants (logFC = 2.09, *p* = .0009, *q* = .054) 7. No association between temperament and presence or relative abundance of *Prevotella* or Lachnospiraceae, when assessed for reverse causation.	1. Insufficient information to estimated dietary intake of fermentable fiber. 2. Relatively small number of cases with elevated behavioral problems. 3. Absence of potential metagenomic or transcriptomic data from which to infer potential mechanisms underlying the observed data.
4. Wang et al. (2020)	None given	Relative abundance at OTU level.	1. Six genera were associated with infant temperament. Bifidobacteria were positively associated with soothability. Cuddliness was negatively correlated with *Hungatella*. 2. Boys and girls showed no significant differences in temperament at 12 months old.	1. This study was a small‐scale test of the associations between infant's gut microbiota and temperament at the age of 12 months. 2. Most infants in the study were taking probiotics. 3. The study did not address the role of diet quantity or quality at the time of stool sample collection, which may account for some of the differences in the associations.
5. Flannery et al. (2020)	Functional beta diversity measured.	Taxonomic and functional composition measures of the microbiome.	1. The quality of caregiver–child relationship moderates the associations between socioeconomic risk and both the structure and functional capacity of the gut microbiome. 2. The quality of caregiver–child relationship moderates the association between measures of behavioral dysregulation and the gut microbiome's functional capacity. 3. Specific gut microbial taxa are associated with socioeconomic risk and behavioural dysregulation.	1. The study was cross‐sectional and therefore it was not possible to determine which child later developed a psychiatric disorder. 2. It was not possible to discern a causal role of the microbiome upon behavioral dysregulation.
5. Flannery et al. (2020)			4. *Bacteroides fragilis* was associated with both higher socioeconomic risk and behavioral dysregulation and reduced levels of aggressive behavior, emotional reactivity, externalizing behavior, sadness, and impulsivity. 5. Butyrate‐producing taxa, specifically *Coprococcus comes* and *Eubacterium rectale*, are positively associated with elevated anxious depression and reduced inhibitory control. 6. *Roseburia inulinivorans* is associated with a decrease in depressive problems.	
6. Kelsey et al. (2021)	Alpha diversity—Shannon Diversity Index and Chao1—was calculated for both taxa and Chao1 functional terms for virulence factors, resistome, and GO terms using the vegan R package.	Both taxonomic and functional relative abundance was used.	1. There were no significant associations between alpha diversity (Shannon‐taxa/Chao1‐taxa) and behavioral temperament measured using the IBQ‐R. 2. A mediation analysis suggests that the relationship between increased alpha diversity and negative emotionality may be mediated by homologous–interhemispheric connectivity. 3. There were no significant indirect effects found for the relationship between taxa diversity and regulation/orienting.	1. The study is limited to one time point in early development. 2. A single fecal sample was collected in the home environment and not in the laboratory environment.
6. Kelsey et al. (2021)	Beta diversity was measured to calculated as the relative abundance in parts per million of each feature used.		4. There was a significant indirect effect found to suggest that the relationship between virulence factor diversity and negative emotionality may be mediated by homologous–interhemispheric connectivity. 5. A similar significant result was found to suggest that the relationship between virulence factor diversity and regulation/orienting may also be mediated by homologous–interhemispheric connectivity through a negative association. 6. Both negative emotionality and regulation/orienting were marked by an enrichment in *Bifidobacterium*. Particularly *B. pseudocatenulatum* was enriched in both the high negative emotionality and high regulation/orienting groups. 7. In a linear model including regulation/orienting, negative emotionality, and surgency as fixed effects, *Thermovibrio guaymasensis* was identified as a significant biomarker for negative emotionality in the unadjusted model.	3. Although rs‐fNIRS was used to measure brain connectivity, as it allows the infant to remain with their mother, it is limited to monitoring activity in superficial structures, and does not allow for measurement of deeper cortical and subcortical structures. 4. By adjusting the model for some confounding factors, some of the association effects are no longer statistically significant. 5. Even though participants were instructed to bring the samples into the laboratory within a 24‐h window, it was not possible to freeze stool samples immediately after collection.

Study 2 used a cluster analysis approach, identifying three distinct community types in infants aged 2.5 months that were related to temperament traits at 6 months of age. Within these clusters, five OTUs presented as the most discriminating and represented the taxa *Veillonella dispar*, *Clostridium neonatale*, *Bacteroides* including *Bacteroides fragilis* (named as Bacteroides cluster), *Enterobacteriaceae*, and *Bifidobacterium*. The cluster dominated by *Bifidobacterium* and *Enterobacteriaceae* presented the highest scores in the temperament trait of regulation and subscales of High‐Intensity Pleasure, Cuddliness, and Duration of Orienting, which is a measure of the time an infant spends paying attention to or interacting with a single object (Putnam et al., 2006). The lowest scores for each of the same trait and subscales were found in *Bacteroides*‐dominant cluster.

Taxonomic composition analysis carried out in Study 5 found (using pairwise comparisons) that *Bacteroides fragilis* was associated with reduced levels of sadness and impulsivity, and increased levels of inhibitory control in children, measured using the CBQ, aged 5–7 years. They identified three known butyrate‐producing taxa, specifically *Coprococcus comes* and *Eubacterium*, that were positively associated with elevated anxious depression and reduced inhibitory control. Interestingly, the third butyrate‐producing bacterium *Roseburia inulinivorans* was associated with a decrease in depressive problems. Using the shotgun metagenomic technique, Study 5 also investigated the functional capacity of the GM. They found that fear was positively associated with both heme/iron biosynthesis and biosynthesis of melatonin metabolized from tryptophan. Tryptophan metabolism was additionally positively associated with impulsivity.

Study 6 used linear discriminant analysis of effect size to identify five microorganisms as potential biomarkers for temperament in infants aged 9–56 days old. Both negative emotionality and regulation/orienting were associated with increased levels of *Bifidobacterium*, specifically increased negative emotionality and regulation/orienting were found in those individuals whose gut microbiome was enriched by *B. pseudocatenulatum*. Further analysis using microbiome multivariate associations with linear models (Maaslin2) found an additional biomarker of *Thermovibrio guaymasensis*, which was associated with negative emotionality.

Finally, in a longitudinal approach, Study 3 assessed reverse causation considering associations between early temperament, measured using a 5‐point Likert scale, and the candidate bacteria, *Prevotella* and *Lachnospiraceae*. These bacteria were established as candidates for further investigation via earlier examination of the link between microbiota composition and risk of elevated behavior problems in 2‐year‐olds. There were no associations found between temperament measured at 1, 6, and 12 months and presence or abundance of either bacterium. Furthermore, the relationship between normalized abundance of *Prevotella* and behavior measured at 2 years was not attenuated by adjusting for temperament.

### 3.6 Associations between covariates, the gut microbiome, and temperament

In addition to the relationship between GM and temperament, several covariates were discussed within four of the five studies. Study 3 did not adjust for covariates in their reverse causation investigation of temperament.

Study 1 focused primarily on differences between genders in GM composition and temperament scores and found that there were significant differences between males and females (see Table 4). Study 2 also found positive associations between Surgency subscales in boys and relative abundance of *Bifidobacterium* OTUs. In addition to gender, Study 2 also considered the potential effects of several covariates, including gestational age, infant age, mode of delivery, breastfeeding status, and antibiotic intake age. Results of adjusted models are presented above. Study 4 investigated gender differences in temperament only and found no significant difference in scores measured on the IBQ‐R. Maternal education level was positively related to the temperament measures of soothability. Furthermore, this study controlled for several covariates in their model including delivery mode, feeding type (breast or formula), and probiotic consumption.

Study 5 investigated covariates of gut‐related history and diet categories using a daily diary of basic food categories that the child ate at breakfast, lunch, and dinner in the week prior to the laboratory visit. Diet was categorized as the average number of days a child's diet contained food in any one of the following categories: grains, vegetables, fruit, meat, other type of protein, dairy, yoghurt (separate than dairy), beans/nuts/seeds, and sugars/fats/oils. In addition, the average number of food categories (diversity in diet) that a child had per day was also measured. They found that 12.5% of the variation in functional composition and 25.3% of the taxonomic composition were explained by these variables.

Study 6 included several covariates and found that Shannon‐taxa diversity was significantly associated with birthweight, income, breastfeeding, gestational age, and head circumference. There were no associations between Chao1‐taxa and any covariates. For functional term diversity, there were significant associations with resistome diversity and income, gestational age, and maternal depression scores. Virulence factor diversity was also significantly associated with income and antibiotics administered at the hospital after birth. Significant associations were also found between the temperament measurement of negative emotionality, and the covariates of infant age and income. The results above present the adjusted models.

## 4 DISCUSSION

The GM composition, diversity, and function and its relationship with the GBA are emerging as an important area of research in understanding the causal pathways of behavioral and mental health problems in later childhood, adolescence, and into adulthood. This systematic review aimed to determine whether there was empirical evidence supporting the relationship between GM diversity and composition and temperament outcomes in children from birth to the age of 6 years 11 months. A total of six articles were identified, each from a unique study sample that examined both the GM and temperament in early childhood.

### 4.1 Findings regarding microbiome diversity

The findings from the studies examining alpha diversity fall into two patterns that are distinguishable by age from birth to 12 months, and 12 months and over. Twelve months of age is a significant time of maturation of the gut microbiota: as the diet moves away from milk‐based to solid food intake, the microbiota moves toward a more diverse composition in healthy individuals. Aatsinki et al. (2019) and Kelsey et al. (2021) both presented results consistent with a tentative pattern of no significant associations between diversity of the microbiota and temperament outcomes before 12 months of age. Kelsey et al. (2021) did, however, find significant indirect associations between alpha diversity of taxa (Shannon and Chao1) and negative emotionality, and alpha diversity indices for functional terms (virulence factors) and both negative emotionality and regulation/orienting when mediated by homologous–interhemispheric neural connectivity. The pattern of this relationship suggests that increased connectivity at this stage of development is an aberrant response, which would not be expected later in childhood when increased alpha diversity would be beneficial. The mechanism underlying this warrants further exploration. For example, do increases in the strength of connectivity at that stage of development reflect delayed maturation of usual brain networks or a response to altered microbiome? Kelsey et al. (2021) compare their findings to previous literature that examined the link between alpha diversity and cognitive performance in infants aged between 1 and 2 years of age (Carlson et al., 2018). However, it is important to note that cognitive performance, behavior, and temperament/personality measure very different aspects of child development, and therefore this limits the conclusions that can be made from comparison of the role of alpha diversity in these differing developmental outcomes.

Findings in children aged over 12 months of age show that higher alpha diversity was associated with Surgency/Extraversion in both males and females and High‐Intensity Pleasure in males. Higher alpha diversity was negatively associated with Effortful Control in females (Christian et al., 2015). Variation in gut microbiota community structure, measured as beta diversity unweighted UniFrac distances, showed that there is a unique community structure associated with the temperament trait Surgency/Extraversion in males and Fear in females (Christian et al., 2015). Interestingly, Surgency/Extraversion in males was associated with both higher alpha and unique beta diversity. In summary, there is a very small amount of evidence to support the idea of a link between GM diversity and temperament. The tentative pattern showing no association between temperament and alpha diversity before 12 months of age should be viewed cautiously due to methodological shortfalls in the papers reviewed. These include differences in microbiota analysis selected (including use of both 16S rRNA [Aatsinki et al., 2019; Christian et al., 2015] and shotgun metagenomics [Kelsey et al., 2021] methods) and limited control of important confounding factors (e.g., environmental factors). Of the three papers evaluating these relationships, two studies were conducted in the United States and one was conducted in Finland, but no mention was given to whether participants lived in rural or urban locations, which has previously been shown to be associated with the diversity and richness of the gut microbiota (Salim et al., 2014; Zuo et al., 2018). Given that only three papers have measured these relationships and there is a lack of overlap in study design and measures, the patterns of findings are not wholly consistent; more research is needed to increase confidence in the absence or existence of any causal relationships and meaning of these tentative associations. It would be premature to draw the conclusion that the pursuit of further investigations of the relationship between microbiome and temperament prior to 12 months of age is not necessary on the basis of the small amount of work in the field to date. More work is required to investigate the temporal and causal relationships between microbiome and temperament in these early months and our review highlights a range of factors that are important to consider for optimal study design in future studies.

### 4.2 Findings regarding microbiome composition

In contrast to measures of diversity, which tell us about the number of different taxa found within the microbiota, and the number of functional differences between them, the composition of the GM allows us to identify specific taxa of interest and how they shape the relationship between the gut microbiota and temperament. When investigating the taxonomic composition of the GM and temperament, we found tentative patterns from the results of the six studies identified. Significant associations between abundance of *Bacteroides* and temperament were found in two studies (Aatsinki et al., 2019; Flannery et al., 2020). Microbiota dominated by *Bacteroides* in 2.5‐month‐olds were associated with lower scores of High‐Intensity Pleasure, cuddliness, and duration of orienting measured at 6 months of age (Aatsinki et al., 2019). Specific associations with increased relative abundance of *B. fragilis*, measured in 5‐ to 7‐year‐olds, were associated with reduced levels of sadness and impulsivity and increased levels of inhibitory control (Flannery et al., 2020). Whilst the results of Aatsinki et al. (2019) and Flannery et al. (2020) appear to contradict each other, it should be noted that the composition of the microbiota undergoes large changes between 2.5 months and 1 year, as solid food is introduced into the diet and the microbiota matures. Thus, cross sectional patterns observed in early infancy may not be predictive of later relationships.

Interestingly two studies (Aatsinki et al., 2019; Kelsey et al., 2021) found significant relationships between *Bifidobacterium* and temperament. Kelsey et al. (2021) found that in children aged between 9 and 56 days of life, higher abundance of *Bifidobacterium* was significantly associated with both high negative emotionality and high regulation/orienting. Aatsinki et al. (2019) found similar results, with higher durations of orienting at 6 months of age in infants whose microbiota was dominated by *Bifidobacterium* at 2.5 months of age. Additionally, they found higher scores of High‐Intensity Pleasure in those children whose microbiota was dominated by *Bifidobacterium* at 2.5 months of age. When combined, the findings of Aatsinki et al. (2019) and Kelsey et al. (2021) allude to a potential link between relative abundance of bifidobacteria and emotional regulation. As this was measured in very early infancy, future research should investigate whether the link between gut microbiota and emotional regulation persists through to later childhood given the large amount of variation and change that occurs during the maturation of the infant gut microbiota. Overall, there is a need for more longitudinal research in this area, which would allow for the mapping of changes to the microbiota, and the impact this can have upon the development of infant temperament.

Flannery and colleagues (2020) found that two butyrate‐producing bacteria, *C. comes* and *Eubacterium rectale* were associated with elevated anxious depression and reduced inhibitory control. Conversely, they also found that another butyrate‐producing bacterium, *R. inulinivorans*, was associated with a decrease in depressive problems. Ruminococcaceae, found to be associated with sociability in boys (Christian et al., 2015), is a family of bacteria also known to produce butyrate. Although these results are somewhat contradictory, these data provide support for the notion that the influence of butyrate‐producing bacteria upon temperament should be an important focus for further investigation. Future research could focus on butyrate‐producing bacteria known to colonize the GM, and their overall role in the relationship between GM and temperament. Furthermore, butyrate‐producing bacteria metabolize complex carbohydrates and dietary fiber and have previously been shown to be beneficial to cognitive function, social behavior, and mental health in animal models (Stilling et al., 2016). Interestingly, the family Rikenellaceae, found to be positively associated with fear in girls (Christian et al., 2015), has been associated with diets high in fat and low in dietary fiber in animal models (Nagano & Yano, 2020). Assessment of the functional composition of the GM indicates that metabolism of tryptophan found in the diet is associated with fear and impulsivity measured on the CBQ (Flannery et al., 2020). Tryptophan is consumed in dairy products, proteins, such as turkey and chicken, and nuts and seeds. It is also found in breast milk and is used in the production of melatonin, which is further associated with mood and depressive state (De Crescenzo et al., 2017; Lanfumey et al., 2013; Srinivasan et al., 2006). However, due to the lack of defined study population and sample size, the quality of the Flannery et al. (2020) paper was judged as poor, and therefore caution exercised in its interpretation. It will be particularly important to replicate such findings before firm conclusions are made. Further investigation should also attempt to probe these relationships by examining the role of diet and the influence this has upon the relationship between GM and temperament.

The main question this review sought to address was whether there was evidence of associations between GM composition and diversity and temperament in children during early childhood. Although there are some interesting patterns emerging, the evidence is still clearly preliminary and only tentative patterns can be discerned. The findings of this review show that replication and extension of existing research is needed in the field of GM in order to unlock more of the potential links with temperament during early childhood. This would then pave the way toward targeted interventions in early childhood that could alter future well‐being.

### 4.3 Limitations of captured studies and the current review

There were several limitations of the studies that may explain some of the variability in findings, including GM factors (e.g., microbiome analysis technique and hypervariable region chosen), study design, and time points analyzed. Of the six studies in this review, two identified that sample size was small. When looking at the quality assessment carried out for all six studies, none of the studies identified power calculations or presented sample size justification, although one study (Kelsey et al., 2021) did provide effect sizes. This is not currently unusual in this field; it is not common practice in GM studies because there is no standard approach for a priori sample size calculation (La Rosa et al., 2012), which can be a major limitation of this type of study.

Additionally, the selection of hypervariable region for analysis is an important part of the GM analysis pipeline. Of the four studies analyzing 16S rRNA, three separate combinations of hypervariable region were selected. Selection of the V4 or V4–V5 regions has been shown to alter or even miss the relative abundance of important taxa in samples taken from the young, such as bifidobacteria species, and substantially increase the abundance of Firmicutes (Alcon‐Giner et al., 2017; Biol‐Aquino et al., 2019). This variation in selection of hypervariable region may contribute to the lack of a distinct pattern emerging between GM composition and temperament. Furthermore, the variety of collection, processing, and analysis pipelines used in the studies contained within this review further impedes the ability to generalize the results between gut microbiota and temperament. The field of GM analysis is also increasingly moving toward a whole genome or shotgun metagenomic approach, which provides both higher resolution and additional functional information (Jovel et al., 2016). Two studies (Flannery et al., 2020; Kelsey et al., 2021) used a shotgun metagenomic approach to investigate the relationship between GM and temperament; however, neither of these two studies primarily focused on the relationship between GM and temperament. Flannery et al. (2020) included several early childhood environmental exposures, such as quality of caregiving and life experiences, and Kelsey et al. (2021) focused on functional neural connectivity and the mediating effect this has upon the relationship between gut microbiome and behavioral temperament. Thus, despite the promise of this technique, there are insufficient data to date that reliably explore the association with temperament.

A further limitation of the studies selected in this review was the study design, which in many cases did not allow for discernment of the causal role of the GM upon temperament. The first year of life is a window of critical development of both the GM and neurodevelopment (Carlson et al., 2018; Knickmeyer et al., 2008; Stewart et al., 2018). Selection of a single measure of both GM and temperament gives only a snapshot of the interaction that is occurring. To discern the causal role of the GM and to measure developmental trajectories, a longitudinal approach with measures taken concurrently for both GM and temperament would be beneficial. Additionally, future studies should carefully consider the role of confounding variables such as diet, gender, and environmental factors known to influence the microbiome.

Finally, regarding the measures of temperament for each study, all studies used a measure that was completed by the mother. Only Aatsinki et al. (2019) identified this as a limitation to their study, stating that choosing maternal reports of temperament may show different results to laboratory‐based assessments as maternal measures of child temperament are known to be influenced by the mother's own temperament and other characteristics (Bayley & Gartstein, 2013). To improve upon this limitation, future studies should consider collecting temperament measures from more than one source, such as additional questionnaires completed by another primary caregivers, or inclusion of laboratory‐based observations in addition to parental/caregiver ratings.

This review had some limitations. First is the limited number of studies included, influenced by the low number of studies examining both the GM and temperament. Another limitation is the heterogeneity in the methodologies used across studies, including the data collection and GM analysis pipeline. Most studies used 16S rRNA techniques; however, all studies varied in hypervariable region selection, library selection, and statistical approach, which resulted in synthesis of the results being more challenging. Overall, there was a lack of overlap between measures and study design, which, in combination with the small number of studies, impedes the generalizability of results.

### 4.4 Future research recommendations

The findings of this review highlight key areas for improvement in future research that investigates the association between GM and temperament in infancy and early childhood.

Development of a standard method to determine sample size and calculate power would vastly improve the field and allow for more consistent and robust GM analysis.

Increased use of shotgun or whole‐genome sequencing approaches would allow assessment of the functional role that species play in the development of the GBA as well as identifying the presence of species within the community.

Future studies should also employ longitudinal approaches that take measurements of GM and temperament both concurrently and in series to establish causal pathways between GM and temperament. This would require careful prospective control for known or theoretically likely confounding variables.

Inclusion of dietary measures in studies of GM and temperament. Temperament in infancy is linked to diet quality (Lipsanen et al., 2020), in particular, consumption of fewer vegetables and increased consumption of sugar‐sweetened drinks and desserts, a dietary pattern associated with lower GM diversity and higher colonization of aberrant species (Martinez et al., 2017). In contrast, animal models have shown that dietary fiber increases abundance of butyrate‐producing bacteria (Zhao et al., 2018). This may highlight the potential for subsequent development of dietary intervention that has relevance to the GM/child temperament association.

Finally, the tentative association between the butyrate‐producing bacteria and temperament appears to be an important one that warrants further investigation.

## 5 CONCLUSION

This systematic review synthesizes current evidence for the relationship between temperament and GM diversity and composition in infancy and early childhood. Several tentative patterns have emerged from this review. First, the direct relationship between alpha diversity and differences in community structure, beta diversity, and temperament is only evident in children over 12 months of age. Second, there is some indication that bacteria that metabolize dietary fiber and complex carbohydrates are important taxa of interest when investigating the relationship between GM and temperament. Finally, from the perspective of temperament, the results indicate that there is a link between variation in the diversity and composition of the GM, and both emotional regulation and fear.

Previous research has generally adopted a cross‐sectional approach, or included only a single measure of GM, which limits the ability to identify causal pathways in the relationship between GM and temperament. To improve this, longitudinal approaches should be adopted using both serial and concurrent measures. Additionally, most research in this area has used a 16S rRNA approach to investigate the composition of the GM. To gain a deeper understanding of the relationship, future research should consider using whole genome methods to understand functional aspects of the GM, and further investigate the potential metabolomic relationship between the GM and temperament.

## CONFLICT OF INTEREST

The authors declare no conflict of interest.
